# Reverse Segond Fracture Associated with Anteromedial Tibial Rim and Tibial Attachment of Anterior Cruciate Ligament Avulsion Fractures

**DOI:** 10.1155/2017/9637153

**Published:** 2017-08-29

**Authors:** Yehia H. Bedeir

**Affiliations:** Orthopedic Surgery Department, El-Hadara University Hospital, University of Alexandria, Alexandria, Egypt

## Abstract

Reverse Segond fracture is an uncommon avulsion fracture of the tibial attachment of the deep portion of the medial collateral ligament of the knee. We report a reverse Segond fracture associated with anterior cruciate ligament tibial avulsion fracture and anteromedial tibial rim fracture. Unlike previous reports, the combination of reverse Segond fracture, anteromedial tibial rim fracture, and anterior cruciate ligament avulsion fracture was not associated with posterior cruciate ligament injury, posterolateral corner injury, or tibial plateau fracture. This new combination of injuries provides better understanding of the mechanisms of ligamentous injuries of the knee and highlights the importance of meticulous assessment of these injuries for accurate diagnosis and subsequent management.

## 1. Introduction

Reverse Segond fracture is an avulsion fracture of the medial proximal tibia, caused by the deep portion of the medial collateral ligament (MCL). It was first described by Hall and Hochman in 1997 [1].

In the few cases reported, reverse Segond fracture has been associated with multiple knee injuries including tibial plateau fractures, medial meniscal root tears, and cruciate ligament tears or avulsion fractures [1–6]. All reported cases having both a reverse Segond fracture and an anterior cruciate ligament (ACL) injury were also associated with posterior cruciate ligament (PCL) injuries or tibial plateau fractures [2, 4, 5]. Anteromedial tibial rim fractures have been associated with posterolateral corner (PLC) and PCL injuries [7–10] or generalized joint laxity [11].

We report a case in which a reverse Segond fracture was associated with anteromedial rim fracture and ACL avulsion fracture, without associated injuries in PCL, PLC, or tibial plateau. We believe this case report provides new insights to deeply understand the mechanisms of ligamentous injuries of the knee.

## 2. Case Presentation

**Figure 1:**
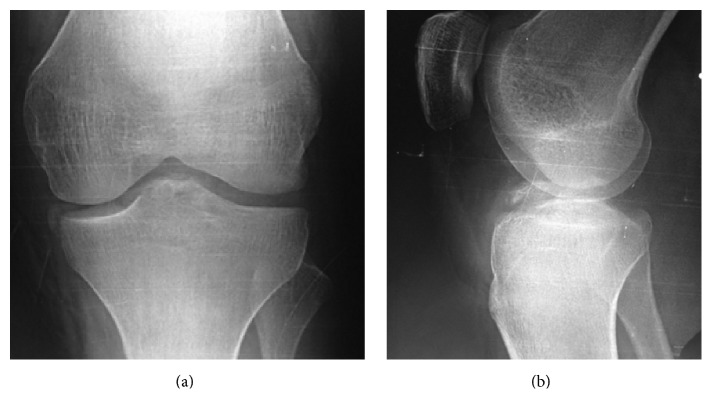
Anteroposterior (a) and lateral (b) preoperative radiographs showing tibial eminence and reverse Segond fractures.

**Figure 2:**
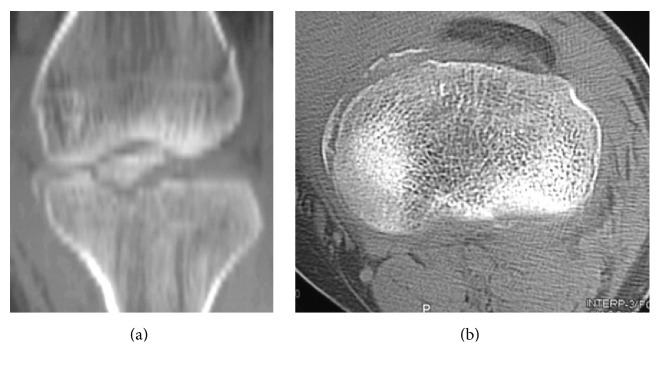
Computed tomography: (a) coronal image showing tibial eminence and reverse Segond fractures and (b) axial image showing reverse Segond and anteromedial rim fractures.

**Figure 3:**
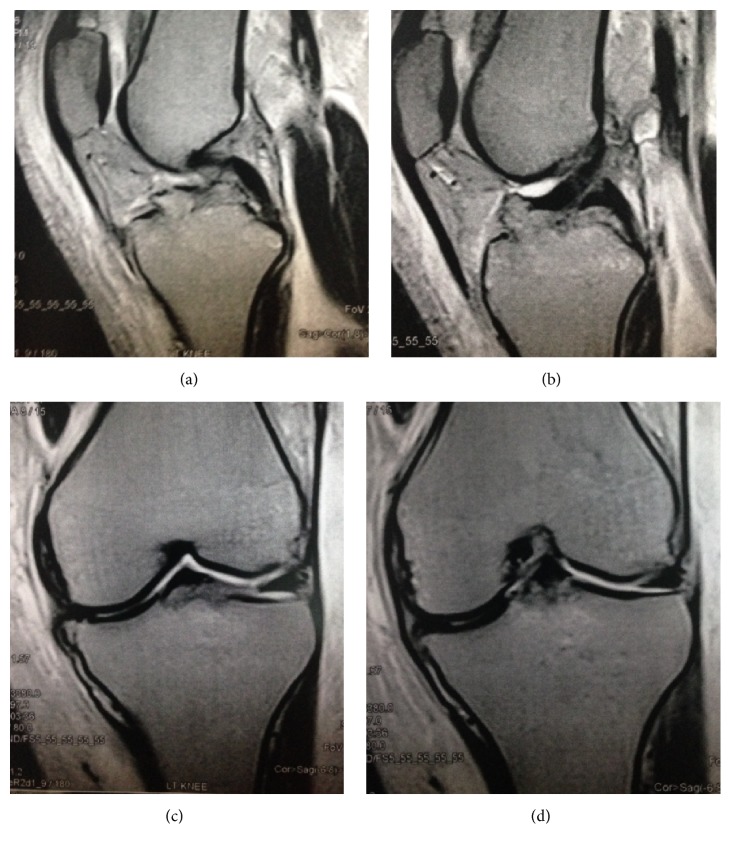
Magnetic resonance imaging showing intact PCL (a), avulsion of the tibial attachment of the ACL (b), reverse Segond fracture (c), and intact superficial MCL (d).

A 19-year-old man, involved in a motorcycle road traffic accident, presented with a painful swollen left knee. The injured knee had never been significantly injured before. According to the patient's description, he hit a pedestrian during driving a motorcycle which made him lose balance and fall to the ground on his left side where his left knee was injured secondary to a direct trauma to the flexed knee, with the motor bike on top potentially causing a valgus moment at the knee. Clinical examination was evident for medial laxity and ACL failure. Tibial eminence fracture and reverse Segond fracture were seen on anteroposterior and lateral radiographs (Figure 1). Computed tomography confirmed the radiographic findings and revealed an anteromedial tibial rim fracture (Figure 2). Magnetic resonance imaging showed avulsion of the tibial attachment of the ACL and intact PCL (Figure 3).

**Figure 4:**
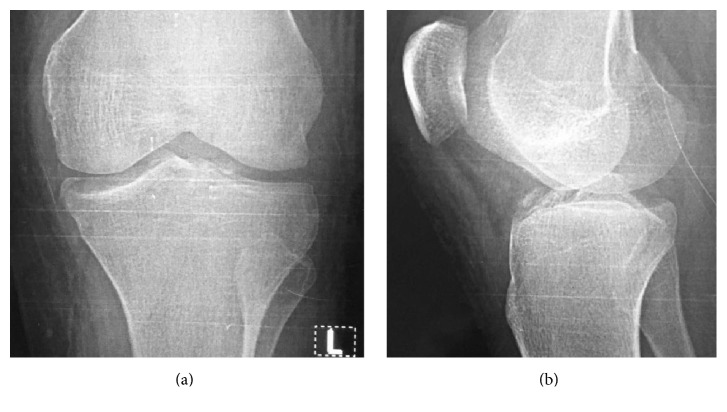
Anteroposterior (a) and lateral (b) postoperative radiographs. Medial and anteromedial rim fractures were repaired using nonabsorbable transosseous sutures. Tibial attachment of ACL was reduced and fixed in place using pullout sutures.

Examination under anaesthesia confirmed ACL and MCL laxity. Intraoperatively, the deep portion of the MCL with its avulsed bony fragment and the anteromedial rim fracture were repaired using nonabsorbable transosseous sutures. Open reduction and fixation of the avulsed ACL were achieved using nonabsorbable pullout sutures (Figure 4). A hinged knee brace fixed in 15° flexion was applied postoperatively. The hinged brace was fixed for the first 2 weeks except for range of motion exercises. The brace was continued for 3 months. Full extension was achieved on postoperative day 1. Active assisted knee flexion exercises were performed after 2 weeks. Weight bearing was allowed after 6 weeks. After 3 months, full range of motion was achieved and strengthening exercises were implemented.

## 3. Discussion

Reverse Segond fracture is an avulsion of the deep capsular component of the MCL. It has been associated with PCL [1, 3, 6] or both PCL and ACL [2, 4] injuries. The few reports describing both cruciate ligament injuries linked the reverse Segond fracture to PCL and not ACL disruption. The presumed explanation in these reports was that the excessive posterior subluxation and external rotation associated with PCL injury, together with valgus angulation, might cause a reverse Segond fracture [1, 4]. However, Peltola et al. [5] reported 10 patients with reverse Segond fractures with only a single case of PCL avulsion among them. They, therefore, postulated that posterior subluxation and external rotation are not necessary to produce a reverse Segond fracture. They also reported three patients with ACL avulsion fractures, in which they linked these ACL injuries with their associated tibial condyle fractures and did not suggest an association of ACL injuries and reverse Segond fractures [5].

Anteromedial tibial rim fractures have been associated with PCL and PLC injuries [7–10]. This avulsion fracture has been described by Cohen et al. [7] as an impingement fracture of the anteromedial rim of the tibial plateau against the medial femoral condyle owing to the posterior translation of the tibia with PCL disruption.

The current case demonstrates three tibial avulsion fractures within the same injury. To our knowledge, this combination of injuries, without tibial plateau fracture or PCL or PLC disruption, has never been reported. The exact mechanism of injury that led to this pattern of avulsion fractures is hard to prove. The potential mechanism of injury that led to that combination of injuries is consistent with the hypothesis of Peltola et al. [5] that valgus angulation is the most essential step to produce a reverse Segond fracture and that posterior subluxation is not necessary. In this patient, valgus angulation was combined with anterior shear force on the proximal end of the tibia with the leg in flexion and external rotation which led to the disruption of ACL, deep portion of the MCL, and anteromedial capsule. These three structures caused tibial avulsion fractures rather than intrasubstance tears.

## 4. Conclusion

We draw attention to the fact that injuries around the knee may result in a spectrum of bony and/or ligamentous disruptions. Vigilance and meticulous assessment of these types of injuries is essential for accurate diagnosis and subsequent management.

## References

[1] Hall F. M., Hochman M. G. (1997). Medial Segond-type fracture: Cortical avulsion off the medial tibial plateau associated with tears of the posterior cruciate ligament and medial meniscus. *Skeletal Radiology*.

[2] Engelsohn E., Umans H., DiFelice G. S. (2007). Marginal fractures of the medial tibial plateau: Possible association with medial meniscal root tear. *Skeletal Radiology*.

[3] Angelini F. J., Malavolta E. A., D'Elia C. O., Pécora J. R., Hernandez A., Camanho G. L. (2007). Fratura avulsão do planalto tibial medial (Segond reverso). *Acta Ortopédica Brasileira*.

[4] Escobedo E. M., Mills W. J., Hunter J. C. (2002). The "reverse Segond" fracture: Association with a tear of the posterior cruciate ligament and medial meniscus. *American Journal of Roentgenology*.

[5] Peltola E. K., Lindahl J., Koskinen S. K. (2014). The reverse Segond fracture: Not associated with knee dislocation and rarely with posterior cruciate ligament tear. *Emergency Radiology*.

[6] Faroug R., Hasan A. (2009). Reverse Segond fracture: A case report. *Injury Extra*.

[7] Cohen A. P., King D., Gibbon A. J. (2001). Impingement fracture of the anteromedial tibial margin: A radiographic sign of combined posterolateral complex and posterior cruciate ligament disruption. *Skeletal Radiology*.

[8] Chiba T., Sugita T., Onuma M., Kawamata T., Umehara J. (2001). Injuries to the posterolateral aspect of the knee accompanied by compression fracture of the anterior part of the medial tibial plateau. *Arthroscopy*.

[9] Bennett D. L., George M. J., El-Khoury G. Y., Stanley M. D., Sundaram M. (2003). Anterior rim tibial plateau fractures and posterolateral corner knee injury. *Emergency Radiology*.

[10] Yoo J. H., Kim E. H., Yim S. J., Lee B. I. (2009). A case of compression fracture of medial tibial plateau and medial femoral condyle combined with posterior cruciate ligament and posterolateral corner injury. *Knee*.

[11] Chanasit P., Sa-ngasoongsong P., Chanplakorn P., Jaovisidha S., Suphachatwong C., Wajanavisit W. (2013). Anteromedial marginal fracture of medial tibial plateau without significant knee ligamentous injury in hypermobility patient: a case report and review of literature. *Orthopedic Reviews*.

